# Analysis and Research on Spectrogram-Based Emotional Speech Signal Augmentation Algorithm

**DOI:** 10.3390/e27060640

**Published:** 2025-06-15

**Authors:** Huawei Tao, Sixian Li, Xuemei Wang, Binkun Liu, Shuailong Zheng

**Affiliations:** 1 Key Laboratory of Grain Information Processing and Control, Henan University of Technology, Ministry of Education, Zhengzhou 450001, China; lilisixian8@gmail.com (S.L.); bkliu@haut.edu.cn (B.L.); zzzzzzls666@gmail.com (S.Z.); 2 Henan Key Laboratory of Grain Storage Information Intelligent Perception and Decision Making, Henan University of Technology, Zhengzhou 450001, China; 3 Institute for Complexity Science, Henan University of Technology, Zhengzhou 450001, China

**Keywords:** spectrogram, speech emotion recognition, data augmentation, cross-entropy loss

## Abstract

Data augmentation techniques are widely applied in speech emotion recognition to increase the diversity of data and enhance the performance of models. However, existing research has not deeply explored the impact of these data augmentation techniques on emotional data. Inappropriate augmentation algorithms may distort emotional labels, thereby reducing the performance of models. To address this issue, in this paper we systematically evaluate the influence of common data augmentation algorithms on emotion recognition from three dimensions: (1) we design subjective auditory experiments to intuitively demonstrate the impact of augmentation algorithms on the emotional expression of speech; (2) we jointly extract multi-dimensional features from spectrograms based on the Librosa library and analyze the impact of data augmentation algorithms on the spectral features of speech signals through heatmap visualization; and (3) we objectively evaluate the recognition performance of the model by means of indicators such as cross-entropy loss and introduce statistical significance analysis to verify the effectiveness of the augmentation algorithms. The experimental results show that “time stretching” may distort speech features, affect the attribution of emotional labels, and significantly reduce the model’s accuracy. In contrast, “reverberation” (RIR) and “resampling” within a limited range have the least impact on emotional data, enhancing the diversity of samples. Moreover, their combination can increase accuracy by up to 7.1%, providing a basis for optimizing data augmentation strategies.

## 1. Introduction

In modern society, emotional speech recognition technology has become a core component of human–computer interaction systems, with widespread applications in fields such as mental health monitoring [1], customer service quality inspection [2], age-appropriate product design [3], and intelligent transportation systems (ITS) [4]. Accurate recognition of emotion not only helps machines to understand users’ emotions but also significantly enhances system intelligence and user experience.

However, the field of speech emotion recognition (SER) is confronted with a serious issue of data sparsity. Taking the commonly used SAVEE [5] and EMODB [6] datasets as examples, the numbers of samples are merely 480 and 800, respectively. In the face of the complex and diverse emotional expressions of human beings, the coverage of these samples is extremely limited. As a result, data augmentation technology has gradually become an important approach to enhancing the generalization ability of models. Existing studies have examined the role of augmentation algorithms in improving model performance; for example, Atmaja et al. [7] pointed out that for text-independent data (covering the scenario where both the speaker and the text are independent), data augmentation methods can generally boost the performance of speech emotion recognition. Other studies [8] have also highlighted the effectiveness of data augmentation techniques in enhancing the performance of speech emotion recognition. Nevertheless, most studies only remain at the perspective of overall performance improvement, evaluating the augmentation effect through metrics such as accuracy, and have not explored the possible impact of augmentation algorithms on emotional expressions. In our previous research work [9], we merely explored the impact of classical acoustic data augmentation methods on model performance, focusing on model construction and basic analysis of these classical methods. We did not provide an in-depth look at how data augmentation techniques affect the emotional data in speech emotion recognition. Moreover, merely understanding the impact on model performance is insufficient for a comprehensive understanding of the operation mechanism of data augmentation techniques in this field.

In speech emotion recognition, the spectral features of speech signals such as fundamental frequency [10,11], formants [12], and their temporal variations [13] also play a crucial role. With the development of deep learning technology, spectrograms have become increasingly important in speech emotion recognition. When distinguishing emotions with the same arousal level, traditional prosodic features such as intonation, intensity, and speaking rate often lead to a high degree of confusion [14]. Spectrograms can more comprehensively capture the spectral characteristics of speech signals, including fundamental frequency, formants, and their temporal variations, thereby achieving significant advantages in recognizing emotional expressions. A.M. Badshah et al. [15] combined spectrograms with deep learning for emotion recognition and demonstrated the effectiveness of this method on multiple datasets. Zhang et al. [16] explored the integration of spectrograms and emotion recognition models and proposed a new architecture. Biswas et al. [17] proposed a model for recognizing emotions from speech data using logarithmic spectrograms and deep convolutional neural networks (CNNs). H. Li et al. [18] introduced a transformer-based model called MelTrans which extracts key clues from speech data by learning the core features and long-range dependencies in Mel spectrograms of speech. However, most current studies merely use spectrograms as visual aids for auxiliary analysis. They neither thoroughly investigate whether data augmentation techniques affect the core spectral features of spectrograms, such as fundamental frequency and formants, nor systematically quantify the specific extent of such impacts.

In response to the above-mentioned research gaps and challenges, this paper systematically evaluates the impacts of five common data augmentation algorithms: adding noise [19,20], time stretching [21], fundamental frequency transformation [22], resampling [23], and reverberation (RIR) [24,25]. We investigate the effects of these techniques on the spectral features of spectrograms and the performance of emotion recognition, then expound on the advantages and limitations of each method. This study is carried out from three dimensions. First, to analyze subjective auditory perception, listening experiments are designed to evaluate the quality of the augmented speech, noise perception, and the intensity of emotional expression. Second, jointly extracting multi-dimensional features such as the average spectral amplitude, fundamental frequency, formant frequency, and time domain duration from the spectrogram allows us to use heatmap visualization to conduct an in-depth exploration of the influence mechanism of data augmentation algorithms on the spectral characteristics of speech signals. Third, we provide an analysis of objective recognition performance. The cross-entropy loss calculated during the training and testing processes is utilized to objectively evaluate the recognition performance of the model. The misclassification patterns within emotion categories are observed through confusion matrices to comprehensively assess the performance of different data augmentation algorithms in emotion recognition tasks. In addition, this paper also explores the combined application of different data augmentation techniques and studies the impact of their synergistic effects on the performance of the model. Through these approaches, this paper aims to reveal the profound impacts of data augmentation on spectral characteristics and emotion recognition performance, providing a scientific basis for the optimization and application of data augmentation techniques in emotional speech processing.

## 2. Related Theory

### 2.1. Spectrogram Generation Principle

The generation of spectrograms is based on the fundamental theories of signal processing. A standard spectrogram is typically obtained by performing a short-time Fourier transform (STFT) on an audio signal, then presenting the results on a linear frequency scale [26]. The specific formulas are provided below.

For an audio signal x(t), the frame division process is carried out first:

(1) $$x_{m}\left(t\right)=x\left(t\right)\cdot w\left(t-mT_{s}\right)$$

where $x_{m}\left(t\right)$ represents the m-th frame, w(t) is the window function, and $T_{s}$ is the frame shift interval. Then, the short-time Fourier transform (STFT) is applied to each frame:

(2) $$X_{m}\left(f\right)=\int_{-\infty }^{\infty }x_{m}\left(t\right)e^{-j2\pi ft}dt.$$

Finally, taking the time (corresponding to different values of *m*) as the horizontal axis and the linear frequency *f* as the vertical axis, the magnitude values $|X_{m}\left(f\right)|$ of the spectrum $X_{m}\left(f\right)$ of each frame are used as the amplitude of each point and the result is presented as a standard spectrogram.

### 2.2. Data Augmentation Algorithms

The accuracy of emotional speech recognition models is highly dependent on the richness of data, and speech data augmentation techniques are commonly used to address the issue of data scarcity. By applying various transformations to speech signals, these techniques can improve the generalization ability of the trained model and enhance its ability to recognize different emotional states [27]. Table 1 lists common data augmentation algorithms and their implementation methods in spectrograms.

## 3. Proposed Method

### 3.1. Subjective Listening Design

**Figure 1:**
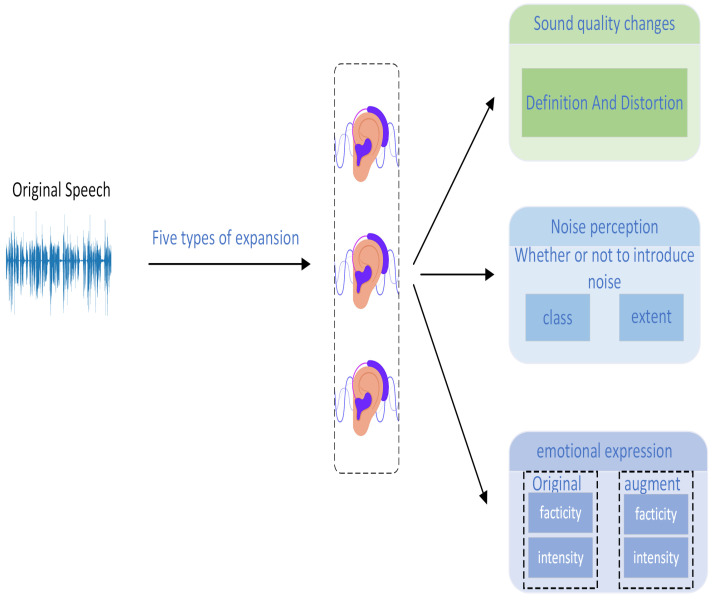
Listening experiment design.

A subjective listening experiment was designed to verify the changes in speech quality and emotional expression resulting from different augmentation algorithms based on human auditory perception. The design of the listening experiment is shown in Figure 1.

Sample Selection: A number of emotional speech segments were randomly selected from the original corpus. Data augmentation was applied with various parameter settings, and representative samples were chosen to ensure multidimensional coverage of emotional categories, speech samples, and augmentation algorithms.

Audience Participation: Three listeners with professional training and expertise in audio analysis who had no hearing impairments were invited to participate in the experiment. They provided detailed feedback on each augmentation algorithm based on their auditory perception. The listeners were asked to describe the following aspects:

- Changes in Audio Quality: The listeners described differences in clarity, distortion, and other audio qualities between the augmented audio and the original audio. They were asked whether the speech still sounded natural and whether there were any unpleasant effects (e.g., blurring, distortion).
- Noise Perception: The listeners noted whether the augmented audio introduced noise, specifying the type of noise (e.g., background noise, buzzing, echoes) and the severity of its impact (mild/significant/severe).
- Changes in Emotional Expression Intensity: The listeners described whether the emotional expression in the augmented audio had changed, such as whether the emotion had been weakened or enhanced. They were also asked whether they could perceive any changes in the authenticity or intensity of the emotion.

### 3.2. Spectral Characteristics Analysis and Feature Extraction of the Spectrogram

**Figure 2:**
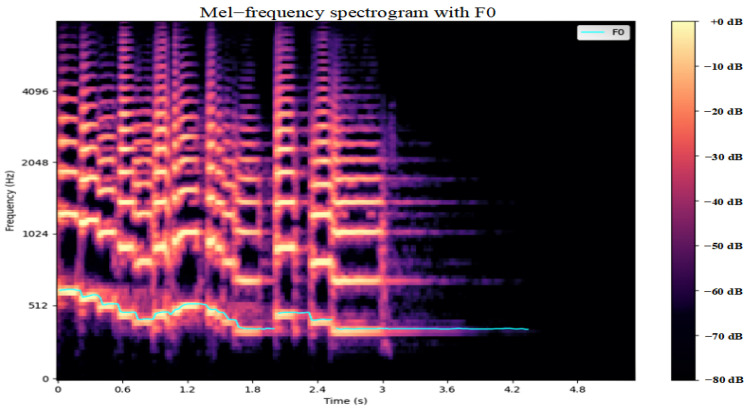
Spectrogram feature analysis.

Spectrograms can display the energy distribution, time domain dynamic characteristics, and frequency variation trends of speech signals. Figure 2 shows information on frequency and time, fundamental frequency (F0), formants, and dynamic changes within the spectrogram. These features provide rich time–frequency information for speech emotion recognition [28]. In research on speech emotion recognition, many scholars have utilized spectrograms to analyze speech features; however, most of these studies are merely at the level of intuitive observation or the extraction of single features. In our previous research work, we evaluated the effect of data augmentation by comparing the visual differences of spectrograms; however, that study lacked a quantitative analysis of the feature changes.

Thus, in this paper we adopt a method of joint extraction and quantitative analysis of multiple features. The Librosa library [29] is used to extract four key features: average spectral amplitude, fundamental frequency, formant frequency, and time domain duration. The specific formulas are as follows:

The short-time Fourier transform (STFT) is used to extract average spectral features:

(3) $$spectra\mathrm{l\_}amplitud\mathrm{e\_}mean=\frac{1}{M\cdot N}\sum_{m=0}^{M-1}\sum_{k=0}^{N-1}\left|X\left(m,k\right)\right|$$

where *M* is the number of frames and *N* is the number of frequency points.

The probabilistic YIN algorithm (PYIN) [30] is used to extract the fundamental frequency:

(4) $$\begin{array}{cc} \; & f_{0}\left(i\right)=\frac{SamplingRate}{period_{i}}for\;each\;frame\;i\\ \; & f0\_mean=\left\{\begin{array}{cc} \frac{1}{N}\sum_{i=1}^{N}f_{0}\left(i\right), & \mathrm{if}\;f_{0}\left(i\right)\;\mathrm{is}\;\mathrm{valid}\\ 0, & \mathrm{if}\;f_{0}\left(i\right)\;\mathrm{is}\;\mathrm{invalid} \end{array}\right. \end{array}$$

where $f_{0}\left(i\right)$ is the fundamental frequency estimated for each frame.

In this study, the second-order linear predictive coding (LPC) method [31] is adopted to extract the formant frequencies of audio signals and the average value of the positive formant frequencies is calculated as a feature.

The signal length calculation used to extract time domain features (time duration) is as follows:

(5) $$tim\mathrm{e\_}duration=\frac{len(audio)}{sr}$$

where len(audio) refers to the number of audio signal samples and sr refers to the sampling rate.

**Figure 3:**
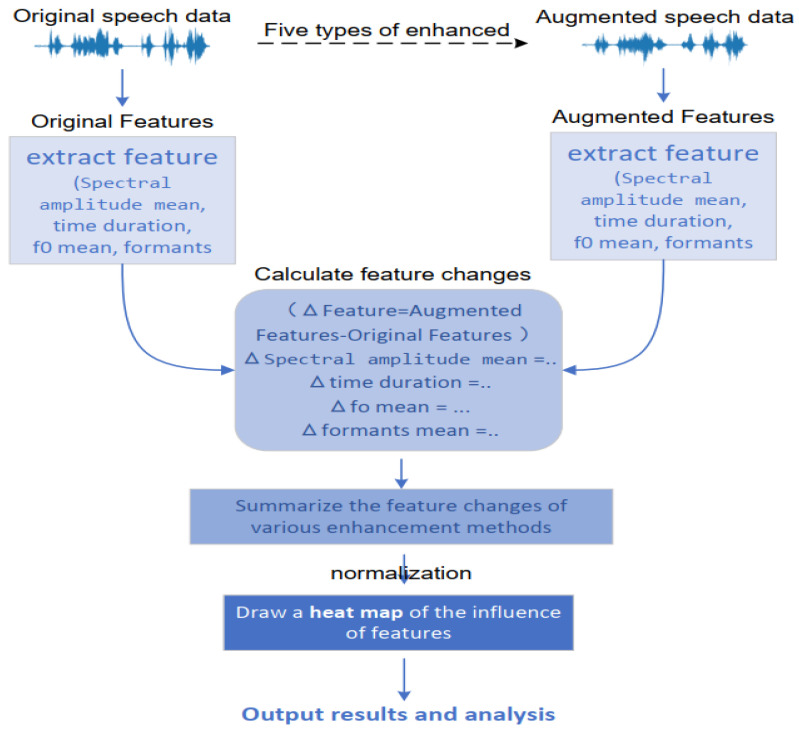
Main design process for feature extraction.

By comparing the original features with the augmented features and visualizing the normalized results through heatmaps, the effects of different augmentation algorithms on these features are demonstrated. Figure 3 shows the main design process for feature extraction.

### 3.3. Objective Emotion Classification Evaluation Design

In recent years, convolutional neural networks (CNNs) have demonstrated remarkable effectiveness in multiple fields, including object detection [32], text classification [33], and ultra-wideband (UWB) communication [34]. However, the application of convolutional neural networks (CNNs) in the field of speech emotion recognition (SER) is mainly attributed to their powerful feature extraction capabilities [35].

When dealing with sequential data, recurrent neural networks (RNNs) are proficient in handling sequential data with temporal dependencies, including text and speech. However, due to the sequential processing characteristics of these networks, they encounter difficulties in capturing global information [36]. In the task of speech emotion classification, emotional information exists not only in the time series of speech but also in the local spatial features of the spectrogram. Given the limitations of RNNs in this regard, CNNs can effectively capture local spatial patterns through convolutional layers, making them more suitable for extracting relevant features.

Numerous studies have confirmed the effectiveness of CNNs in speech emotion recognition. For example, ref. [37] demonstrated through experiments that a model constructed using CNNs can accurately recognize emotions in speech and achieve a high accuracy rate. In [38], the authors successfully applied CNNs to the task of speech emotion recognition. These research findings not only showcase the excellent performance of this method when dealing with actual speech data but also further validate the feasibility and advantages of CNNs in this field.

In view of these previous results, for this paper we select a model in which the feature extractor and the classifier are based on the CNN architecture (see Table 2 and Table 3). Our aim is to conduct a more in-depth evaluation of the impact of data augmentation on the effect of emotion classification.

**Figure 4:**
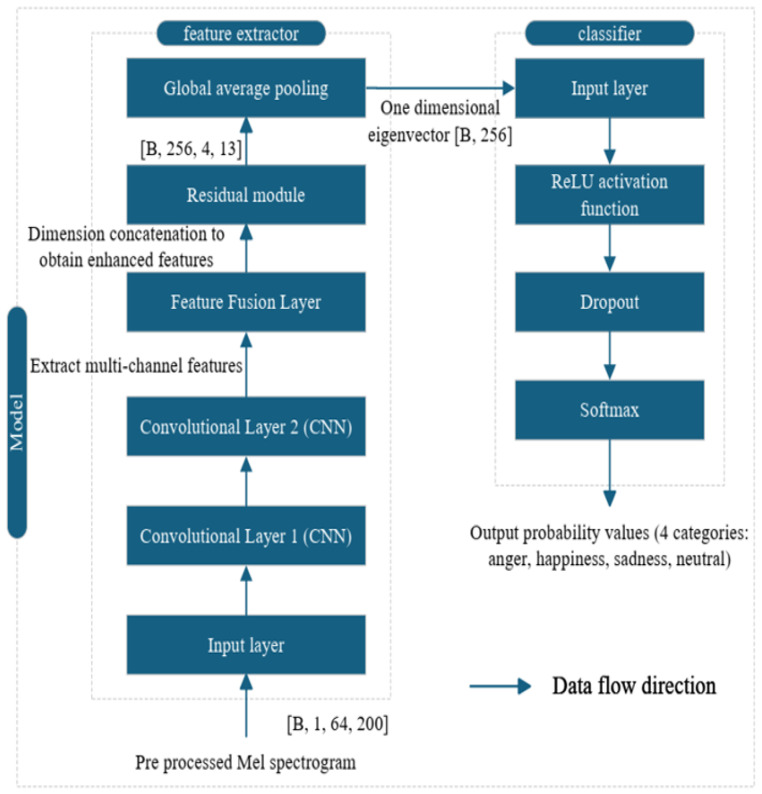
Working process of the feature extractor and classifier.

During the training phase, the feature extractor and the classifier work closely together, as shown in Figure 4. The feature extractor employs a CNN to process the input speech signal. Selecting a relatively small kernel size is beneficial for capturing fine-grained local features, and the combination of two different kernel sizes enables the extraction of features from spectrograms at different scales. Through global average pooling, the high-dimensional feature map is transformed into a one-dimensional feature vector, which is then fed into the classifier part for further processing. For the classifier, the input layer receives the one-dimensional feature vectors from the feature extractor. The first fully connected layer utilizes the ReLU activation function and the dropout mechanism. The ReLU function can effectively alleviate the problem of gradient vanishing and accelerate the convergence speed of the network [39]. Dropout enhances the generalization ability of the model and prevents overfitting to the training data during the training process [40], preparing for the second fully connected layer to output the probability distribution of emotional categories. These two fully connected layers are set up to gradually adjust the feature dimensions and extract more discriminative features to suit the final emotion classification task.

In the process of constructing current emotion models, the selection of optimization objectives is of utmost importance. It is worth noting that the cross-entropy loss is widely adopted as the optimization objective [41,42]. During the training process, this loss function plays a dual critical role; not only does it evaluate the performance of the model by measuring the difference between the predicted probability distribution and the true label distribution [43], it also effectively alleviates the problem of class imbalance in emotion classification tasks. Specifically, in each training iteration, the model first makes predictions on the input data based on its current parameters. Then, the cross-entropy loss value is calculated to reflect the difference between the predicted distribution and the true distribution. Gradients are computed through backpropagation to guide the optimizer (such as Adam) to update the parameters of the model, thereby reducing the loss and improving the classification performance.

In the evaluation phase, the classification results are visualized through a confusion matrix to observe the misclassification of different emotional categories, thereby quantifying the specific impact of data augmentation methods on emotion recognition performance. In addition, metrics such as the weighted accuracy (WA), F1-score, and precision [44] are introduced to comprehensively evaluate the classification performance of different data augmentation methods.

## 4. Experiment Analysis

### 4.1. Experimental Environment and Dataset

Our experiments were conducted on a Windows 10 operating system with the following hardware configuration: NVIDIA GeForce RTX 3060 Ti GPU, CUDA version 12.2, and the Python 3.9 development environment. The parameter settings for the augmentation algorithms are listed in Table 4.

The IEMOCAP (Interactive Emotional Dyadic Motion Capture) dataset [45] was chosen for the experiment. IEMOCAP contains data recorded from ten actors during dyadic (two-person) conversations, with five sessions per conversation and two participants per session. The dataset consists of a total of 10,039 utterances. For this study, the experiment followed the methodology used in existing literature, focusing on four emotion categories: “Anger”, “Sadness”, “Happiness”, and “Neutral”. Samples from the “Excitement” category were merged into the “Happiness” category.

In Section 3.3, the training process used the Adam optimizer (learning rate: 0.001, weight decay: 0.000001) and the cross-entropy loss function. The batch size was set to 64 and the model was trained for 50 epochs with five-fold cross-validation for model optimization.

The five data augmentation algorithms listed in Table 4 were independently applied to the training data, and the model performance was evaluated on the test set.

### 4.2. Subjective Auditory Perception Analysis

Based on the feedback from the listeners, the impact of each augmentation algorithm is summarized in Table 5. Among them, the time stretching algorithm stands out particularly in terms of its impact on emotional expression. After being processed by the time stretching data augmentation method, the speech originally conveying a sense of “sadness” was mistakenly judged by the listeners as expressing “cheerfulness” or “anger”. This phenomenon fully reveals the severe interference of this algorithm with the conveyance of emotional information in speech, causing it to deviate from its original emotional tone.

### 4.3. Impact of Augmentation Algorithms on Spectrogram Spectral Characteristics

**Figure 5:**
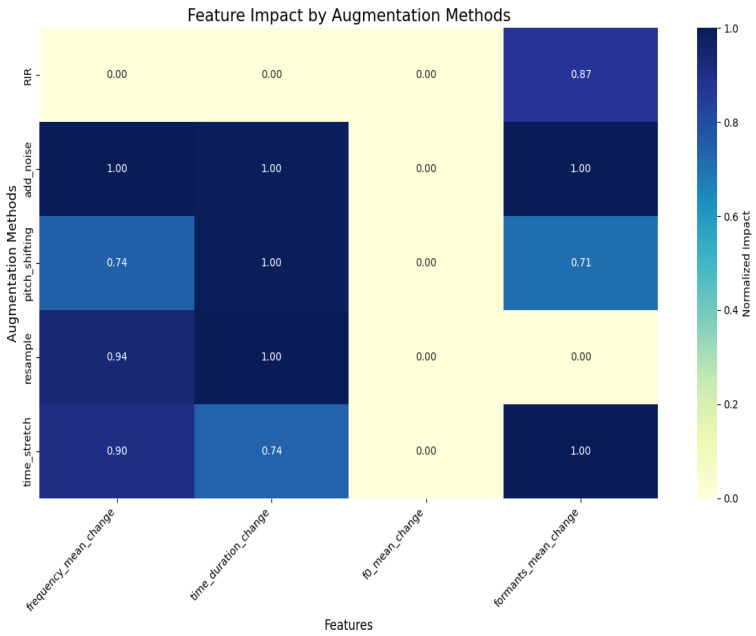
Evaluation results for the impact of augmentation algorithms on spectrogram features.

To analyze the fundamental reasons that listeners misjudged “sadness” as “happiness”, we provide a visual analysis of how different data augmentation techniques affect the characteristics of spectrograms, aiming to reveal the acoustic factors that lead to these perceivable emotional changes. The results are illustrated in Figure 5, which presents the normalized evaluation outcomes of the effects of these algorithms on spectrogram features, encompassing average frequency, fundamental frequency (F0), formants, and temporal characteristics. The comparison yields several observations. First, reverberation (RIR) significantly affects the formant features, causing blurring in the spectrogram and reducing the clarity of emotional expression. Noise addition (add_noise) affects both frequency and temporal features, slightly decreasing audio clarity and making emotional expression less distinct. Pitch Shifting (pitch_shifting) causes subtle changes in both frequency and temporal features, which may lead to confusion between the “Anger” and “Sadness” emotion categories. Resampling (resample) has a considerable impact on frequency and temporal features, but minimal effect on the fundamental frequency and formants. Emotional expression is only mildly disrupted. On the other hand, time stretching (time_stretch) significantly affects frequency and formant features, weakening the naturalness of emotions. It flattens “Happiness” and diminishes the layered quality of “Sadness”.

### 4.4. Objective Emotion Classification Evaluation Results Analysis

**Figure 6:**
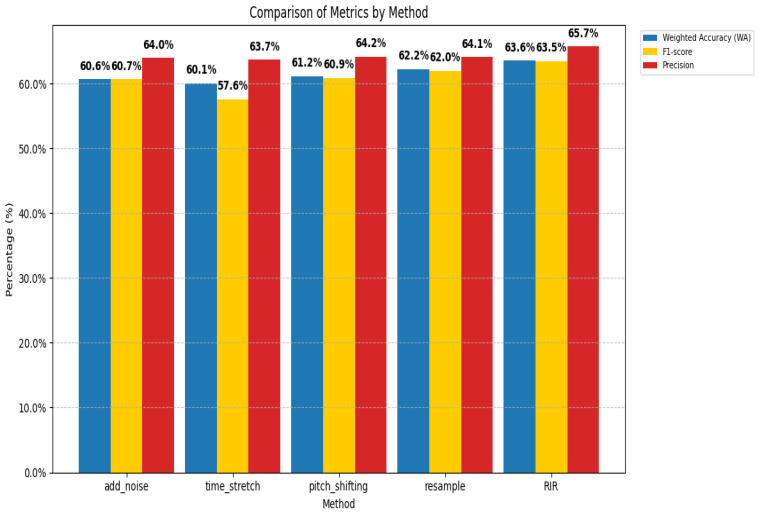
Comparison of model performance metrics under different data augmentation methods.

The above analysis focuses on examining the impact of data augmentation algorithms on spectrogram features from subjective and visual perspectives. To comprehensively evaluate the influence of these algorithms on model performance, we conducted objective evaluation experiments. In a comparison of performance metrics (Figure 6), including weighted accuracy (WA), F1-score, and precision, the reverberation (RIR) method outperformed all others across all metrics, demonstrating optimal overall performance and stability in emotion classification tasks, making it suitable for speech emotion recognition scenarios. The resampling method ranked second, with slightly lower metrics than reverberation. Its stable performance and simplicity make it another viable option. In contrast, noise addition and pitch shifting showed moderate performance but were inferior to the top two methods. The time stretching method exhibited the lowest performance, particularly in terms of the F1-score, indicating a significant negative impact on classification performance which makes it unsuitable as a primary choice. Thus, reverberation is the best choice, followed by resampling.

**Figure 7:**
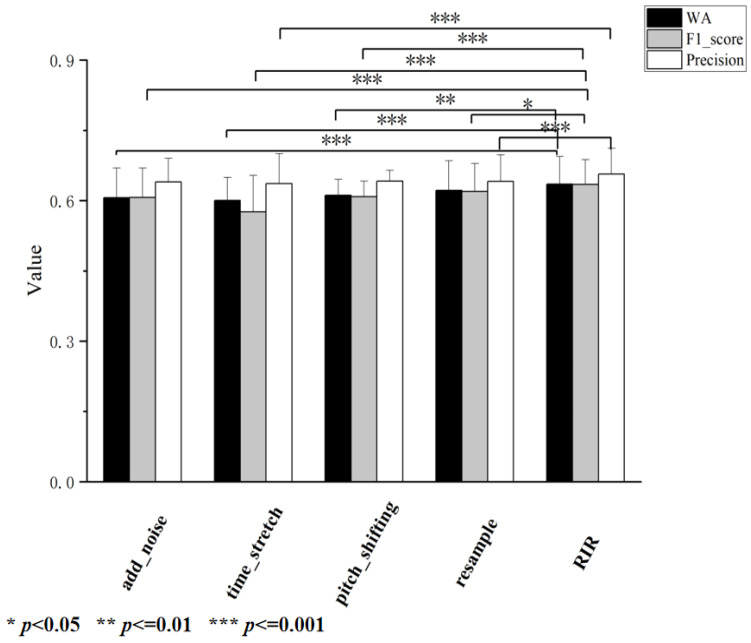
Statistical significance analysis.

To further validate the reliability of the performance differences among different methods, we conducted a statistical significance analysis on the performance of the reverberation algorithm (RIR) compared with the other four algorithms across the three performance metrics of weighted accuracy, F1-score, and precision using the *T*-test method. *T*-tests are used to determine whether there is a significant difference between the means of two groups of data. It calculates the t-statistic and t-distribution, based on which it determines the probability (*p*-value) of observing the current or more extreme data differences under the null hypothesis (i.e., there is no difference between the means of the two groups of data). The results are shown in Figure 7.

- Weighted Accuracy (WA): When compared with the reverberation (RIR) algorithm, the significance markers for the noise addition and time stretching methods are ***, indicating that their *p*-values are less than 0.001 compared to reverberation in terms of the WA metric. This indicates that there is an extremely significant difference between these methods and reverberation in improving the weighted accuracy, with the difference in effects unlikely to be caused by random factors. The pith shifting method is marked with **, indicating that its *p*-value is less than 0.01 and there is a significant difference compared to reverberation.
- F1-Score: The significance markers for the noise addition, time stretching, and pitch shifting methods are ***. This indicates that *p* < 0.001 compared with reverberation, showing an extremely significant difference. The resampling method is marked with *, indicating that *p* < 0.05, meaning that there is a certain degree of significant difference compared to reverberation. This shows that the differences in the effects of these methods on the F1-score compared to reverberation are not accidental.
- Precision: The noise addition method is marked with **, indicating that its *p*-value is less than 0.01 and there is a significant difference compared to reverberation. The time stretching and resampling methods are marked with *** for *p* < 0.001, showing an extremely significant difference compared to reverberation. This reflects that there are obvious differences between these methods and reverberation in terms of precision performance and that these differences are caused by the methods themselves rather than random factors.

The results of the statistical significance analysis indicate that there are real and varying degrees of performance differences between the reverberation algorithm and the other four algorithms across all performance metrics. This provides a quantitative basis for our previous conclusions regarding the performance of different methods and strongly supports the view that reverberation is the best choice among the five data augmentation methods.

**Figure 8:**
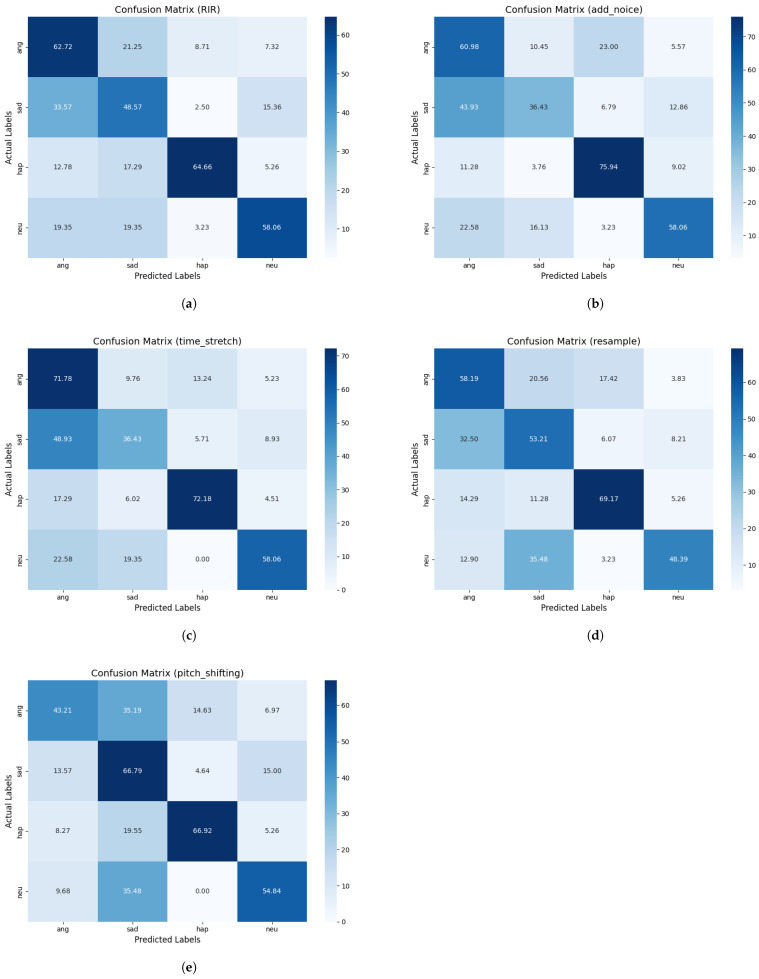
Emotion classification confusion matrix: (**a**) reverberation impulse response (RIR), (**b**) noise addition (add_noise), (**c**) time stretching (time_stretch), (**d**) resampling (resample), (**e**) pitch shifting (pitch_shifting).

To identify the reasons for the performance differences caused by different data augmentation methods, we visualize the confusion matrix in Figure 8 to observe the misclassification distributions of the different methods. Among the tested algorithms, reverberation (RIR) demonstrated the best performance in classifying the “Anger” and “Happiness” emotion categories, with relatively low misclassification rates. In contrast, time stretching caused the most severe interference with the classification of “Sadness” and “Anger” emotions, leading to significant performance degradation.

Table 6 further elaborates the classification performance of each method. The reverberation method not only has the highest accuracy but also maintains the integrity of emotional categories. In contrast, the time stretching method has the greatest negative impact on emotion classification. The main reason for this is that it blurs the boundaries between “Sadness” and “Anger”, which also explains its poor performance. The noise addition, pitch shifting, and resampling methods perform stably when classifying the “Happiness” emotion but cause a great deal of interference when distinguishing between the “Neutral” and “Sadness” emotions. This blurring of the boundaries between the “Neutral” and “Sadness” emotions ultimately leads to the inferior performance of these methods compared to the reverberation method.

When speech processed with the five data augmentation methods is classified by the model, the misclassification rates of “Happiness” and “Anger” are generally low, while those of “Sadness” and “Anger” are relatively high. The reason for this is that “Happiness” and “Anger” are emotions with high arousal levels and obvious characteristic contrasts. The former is characterized by high pitch, fast speaking speed, and a positive rhythm in speech, while the latter features a low pitch and strong intonation changes. These significant characteristic differences make it easier for the model to distinguish between them, reducing the classification error rate. In contrast, “Sadness” and “Anger” both belong to negative emotions and have a moderate arousal level, resulting in many overlapping speech features. For example, the low volume and slow speaking speed of “Sadness” are similar to the speech performance of “Anger” when it is suppressed. These similarities make it highly likely for the model to make misclassifications during emotional classification.

In summary, reverberation not only enhances the features in speech that distinguish between the “Anger” and “Happiness” categories, it also avoids excessively interfere with the discrimination of other emotion types, thereby achieving the best overall classification performance. For these reasons, reverberation is the optimal choice among these five data augmentation methods. Although resampling is less effective than reverberation in distinguishing certain emotions, it demonstrates relatively stable performance in classifying the “Happiness” category and can improve the model’s performance, ranking second as a result. On the other hand, the time stretching method causes the most significant classification interference, especially at the boundaries of emotion categories such as “Sadness” and “Anger”, which greatly increases the misclassification rate and significantly reduces the classification performance. Thus, this method should be avoided possible in practical applications.

**Figure 9:**
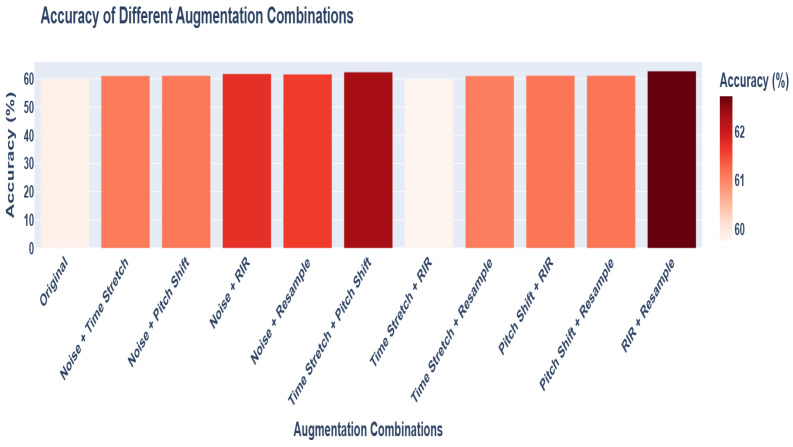
Impact of strategies combining different data augmentation methods on model performance.

Although the individual advantages of resampling and reverberation impulse response (RIR) have been confirmed, the potential synergistic effects that might be generated by the combination of different data augmentation methods still remain to be studied. To address this issue, we systematically combined various augmentation techniques, constructed strategies with multiple different combinations, and evaluated their impacts on the performance of the model, with the results shown in the Figure 9. The results indicate that the combination of resampling and RIR outperforms other combination methods, which verifies the synergistic effect of these two methods in enhancing data diversity and model generalization ability.

In order to further confirm the effectiveness of the resampling and reverberation augmentation techniques and demonstrate the superiority of the proposed model, the performance of the classic network models (ResNet18, VGG16, GoogleNet, DenseNet) and the model composed of a feature extractor and a classifier was compared before and after the application of these two data augmentation techniques, using the weighted accuracy (WA) as the key indicator. This experiment strictly adhered to the settings outlined in Section 4.1, and all models adopted a unified training strategy to ensure the reliability and comparability of the results. The experimental results are shown in Table 7. In benchmark models such as ResNet18, VGG16, and GoogleNet, the application of the resampling and RIR algorithms significantly improved the weighted accuracy (WA), preliminarily verifying the effectiveness of these data augmentation techniques in enhancing the performance of classic network models. After integrating these two augmentation techniques, the weighted accuracy of the model increased by 7.10%, fully highlighting the unique advantage of this model in adapting to data augmentation techniques.

Meanwhile, the proposed model algorithm was compared with several mainstream algorithms on the IEMOCAP dataset, with the results shown in Table 8. The experimental results show that compared with the mainstream algorithms, the weighted accuracy index of the model algorithm has a maximum improvement of 5%, significantly outperforming other algorithms.

In conclusion, the experimental results verify the effectiveness of the research findings from two aspects. First, this experiment further confirms that the resampling and RIR augmentation techniques described in this paper can effectively improve the model performance in speech emotion recognition tasks. On the other hand, it highlights that the model has a higher performance ceiling and stronger adaptability compared to traditional network models after combining the resampling and RIR data augmentation techniques, providing a new reference direction for data augmentation and model optimization in the field of speech emotion recognition.

## 5. Conclusions

This study provides a comprehensive evaluation of the effects of five common data augmentation algorithms (noise addition, time stretching, pitch shifting, resampling, and reverberation) on emotion recognition. The experimental results indicate that reverberation is the most effective augmentation method. It significantly improves sample diversity with minimal interference to emotional features, leading to relatively balanced classification performance. On the other hand, time stretching has the most significant negative impact on emotion boundaries, particularly in classification of the “Sadness” category, where it severely disturbs classification accuracy and significantly reduces overall classification performance. Through experiments conducted across three dimensions, we observed notable performance differences between the various data augmentation methods in terms of emotion recognition, offering valuable insights for optimizing future emotion recognition models.

Notably, in our experiments exploring the combinations of different data augmentation techniques, the combination of resampling and reverberation demonstrated a significant synergistic effect, not only enhancing the diversity of data but also significantly improving the generalization ability of the model. Performance improvement can be achieved without relying on the transformation of any network model. This finding further confirms the potential of combining multiple data augmentation methods for optimizing emotion recognition models.

Based on the results of this study, future research directions can be concentrated on exploring adaptive data augmentation strategies. Through such strategies, appropriate data augmentation methods can be adaptively selected according to different emotional characteristics and model requirements so as to improve efficiency and further optimize the performance of emotion recognition. At the same time, during the process of data augmentation for the original speech data, it is highly likely that the augmented data may introduce new noise or distort the original emotional information, either due to environmental factors or the characteristics of the algorithm itself. Therefore, methods for reducing noise interference are also worthy of more in-depth research, which will help reduce the negative impact of noise on emotion recognition models.

## Figures and Tables

**Table 1:** Implementation methods of data augmentation algorithms in spectrograms.

Data Augmentation Algorithm	Implementation in Spectrograms
Time Stretching	Modifies the time axis of the spectrogram to adjust speech speed or rhythm
Noise addition	Adds background noise or environmental noise to the spectrogram
Pitch Shifting	Adjusts the amplitudes and phases of frequency components to change the frequency distribution, simulating changes in pitch or fundamental frequency
Reverberation (RIR)	Adds reverb in the time domain to simulate different spatial environments, making the frequency distribution in the spectrogram more complex and blurring the distinct frequency boundaries
Resampling	Adjusts the frequency resolution of the spectrogram to simulate speech under different sampling conditions

**Table 2:** Feature extraction structure.

Component	Description
Input Layer	Receives the preprocessed Mel spectrogram with an input size of [B, 1, 64,200]
Convolutional Layer	Two convolutional operations are performed to extract multi-channel features, with kernel sizes of (10, 2) and (2, 8)
Feature Fusion	The convolutional results are concatenated along the channel dimension to obtain an enhanced feature representation
Residual Module	Multi-layer residual modules are applied to progressively enhance the feature representation, with the final output size of [B, 256, 4, 13]
Global Average Pooling	Converts the high-dimensional feature map into a one-dimensional feature vector, with an output size of [B, 256]

**Table 3:** Classifier structure.

Component	Description
Input Layer	Receives the one-dimensional feature vector output from the feature extractor, with size [B, 256]
Fully Connected Layer 1	Maps the features to a 64-dimensional space, using ReLU activation and Dropout to prevent overfitting
Fully Connected Layer 2	Outputs the probability distribution over the emotion categories (4 classes: Anger, Happiness, Sadness, Neutral) using Softmax

**Table 4:** Data augmentation parameters.

Data Augmentation Algorithm	Parameter Settings	Fixed Parameters
Time Stretching	[0.8, 1.2, 1.5, 1.8, 2.0]	——
Noise addition	Signal power $p_{signal}=\frac{1}{N}\sum_{i=1}^{N}x_{i}^{2}$; Noise coefficient $k=\sqrt{\frac{p_{signal}}{10^{\frac{SNR}{10}}}}$; Noisy speech $x_{noisy}=x+k\cdot \varepsilon$ ε∼N(0, 1)	SNR=120 dB
Resampling	Sampling rate: 20,000, First resample from 16,000 to 20,000, then resample from 20,000 back to original sampling rate	Original audio sampling rate: 16,000 Hz
Pitch Shifting	Pitch shift steps: [−6,−3,0,3,6] per octave (semitone): 12	——
Reverberation (RIR)	Source positions: [[1, 1, 1.75], [5, 4, 1.75], [9, 7, 1.75]] Microphone positions: [[0.5, 0.5, 1.2], [4, 3, 1.2], [8, 6, 1.2]]	3D space dimensions: [10, 8, 3.5]

**Table 5:** Listening experiment results.

Augmentation Algorithm	Listener’s Audio Quality Evaluation	Listener’s Noise Perception	Listener’s Description of Emotional Expression Changes
Original Audio	Clear, natural, no distortion	No noise	Emotional expression is clear and unchanged, with the best speech quality
Noise addition	Slightly blurred, mild distortion	Increased background noise (slight noise)	The noise makes the details of emotional conveyance less clear
Time Stretching	Change in speech rate, slight distortion	No noticeable noise	Excessively fast change in speaking speed will lead to the original sad emotion being misjudged as cheerful
Resampling	Audio slightly distorted, frequency variation	No noticeable noise	Distortion affects emotional transmission, especially noticeable in intense emotions (e.g., anger)
Pitch Shifting	Timbre change, good clarity	No noticeable noise	Timbre changes cause subtle emotional differences, especially confusion between anger and sadness
Reverberation (RIR)	The audio has reverberation and is slightly blurry	Slight noise	The speech overlaps, affecting the clear conveyance of emotions

**Table 6:** Analysis of evaluation results.

Augmentation Method	Good Recognition Performance	High Misclassification Rate	Other Observations
Time Stretching	“Anger” (71.78%) and “Happiness” (72.18%)	“Sadness” (48.93% misclassified as “Anger”)	“Neutral” performs poorly, with blurred boundaries, significantly reducing model performance
Reverberation(RIR)	“Anger” (62.72%) and “Happiness” (64.66%)	“Sadness” (33.57% misclassified as “Anger”), “Neutral” (19.35% misclassified as “Sadness”)	Overall stability is better than other methods
Noise Addition	“Happiness” (75.94%)	“Sadness” (43.93% misclassified as “Anger”)	Suitable for increasing data diversity but has a significant impact on boundaries
Pitch Shifting	“Sadness” (66.79%) and “Happiness” (66.92%)	“Anger” (35.19% misclassified as “Sadness”)	“Anger” and “Sadness” confusion is significant
Resampling	“Happiness” (69.17%)	“Anger” (20.56% misclassified as “Sadness”), “Neutral” (35.48% misclassified as “Sadness”)	“Neutral” performs worst, with severe confusion

**Table 7:** Comparison results of performance improvements for different network models on the IEMOCAP dataset.

Method	Baseline	Baseline+RIR+Resample	The Extent of Performance Improvement
Network	WA(%)	WA(%)	(%)
ResNet18 [46]	60.28	62.39	+2.11
VGG16 [47]	52.58	60.94	+8.36
GoogleNet [48]	56.52	59.26	+2.74
DenseNet [49]	59.28	63.32	+4.04
Ours	60.64	67.74	+7.10

**Table 8:** Comparison results between the proposed algorithm and the mainstream algorithms on the IEMOCAP dataset.

Methods	WA(%)
DAMIF [9]	66.51
ADAN+SVM [27]	64.78
WADAN+SVM [27]	63.82
Speech synthesis [50]	66.06
COPYPASTE [51]	63.78
Generative noise [52]	62.74
Ours	67.74

## Data Availability

Publicly available datasets were analyzed in this study. The data can be found at: [IEMOCAP] [https://sail.usc.edu/iemocap/] (accessed on 29 December 2022).
